# Case–control association study of congenital heart disease from a tertiary paediatric cardiac centre from North India

**DOI:** 10.1186/s12887-023-04095-x

**Published:** 2023-06-15

**Authors:** Prachi Kukshal, Radha O Joshi, Ajay Kumar, Shadab Ahamad, Prabhatha Rashmi Murthy, Yogesh Sathe, Krishna Manohar, Soma Guhathakurta, Subramanian Chellappan

**Affiliations:** 1 Sri Sathya Sai Sanjeevani Research Foundation, NH-2, Delhi-Mathura Highway, Baghola, Haryana, District Palwal Pin- 121102 India; 2 Present address Sri Sathya Sai Sanjeevani Research Foundation, Kharghar, Navi Mumbai- 410210, Maharashtra, India; 3 Sri Sathya Sai Sanjeevani Centre for Child Heart Care and Training in Paediatric Cardiac Skills, Navi Mumbai Maharashtra, India; 4 Sri Sathya Sai Sanjeevani International Centre for Child Heart Care & Research, NH-2, Delhi-Mathura Highway, Baghola, District Palwal Haryana, Pin 121102 India

**Keywords:** Congenital Heart Disease, North India, GWAS variants, Case–control association

## Abstract

**Background:**

Congenital Heart diseases (CHDs) account for 1/3rd of all congenital birth defects. Etiopathogenesis of CHDs remain elusive despite extensive investigations globally. Phenotypic heterogeneity witnessed in this developmental disorder reiterate gene-environment interactions with periconceptional factors as risk conferring; and genetic analysis of both sporadic and familial forms of CHD suggest its multigenic basis. Significant association of de novo and inherited variants have been observed. Approximately 1/5th of CHDs are documented in the ethnically distinct Indian population but genetic insights have been very limited. This pilot case–control based association study was undertaken to investigate the status of Caucasian SNPs in a north Indian cohort.

**Method:**

A total of 306 CHD cases sub-classified into *n* = 198 acyanotic and *n* = 108 cyanotic types were recruited from a dedicated tertiary paediatric cardiac centre in Palwal, Haryana. 23 SNPs primarily prioritized from Genome-wide association studies (GWAS) on Caucasians were genotyped using Agena MassARRAY Technology and test of association was performed with adequately numbered controls.

**Results:**

Fifty percent of the studied SNPs were substantially associated in either allelic, genotypic or sub-phenotype categories validating their strong correlation with disease manifestation. Of note, strongest allelic association was observed for rs73118372 in *CRELD1* (*p* < 0.0001) on Chr3, rs28711516 in *MYH6* (*p* = 0.00083) and rs735712 in *MYH7* (*p* = 0.0009) both on Chr 14 and were also significantly associated with acyanotic, and cyanotic categories separately. rs28711516 (*p* = 0.003) and rs735712 (*p* = 0.002) also showed genotypic association. Strongest association was observed with rs735712(*p* = 0.003) in VSD and maximum association was observed for ASD sub-phenotypes.

**Conclusions:**

Caucasian findings were partly replicated in the north Indian population. The findings suggest the contribution of genetic, environmental and sociodemographic factors, warranting continued investigations in this study population.

**Supplementary Information:**

The online version contains supplementary material available at 10.1186/s12887-023-04095-x.

## Background

Congenital Heart Defect (CHD) is common structural abnormality occurring at the time of foetal development. Limited information is available on the exact mechanism of CHD pathogenesis. It affects 9 in 1000 live births globally [1]. This corresponds to 17% of the world CHD load from India [2], yet meagre genetic information available for the disease in the country. In early gestation, incidence is even higher as certain CHDs are complex and have been shown to result in foetal demise [3]. Septational abnormalities account for half of the cardiac congenital defects ranging from nonpathological to lethal [4]. Cardiac development is a complex process and requires intricate coordination of several molecular events for eventual normal structure and function of the heart. Any error/s in these steps result in pathogenic remodelling of heart [5]. Chromosomal aneuploidies like Trisomy 21, 18 and 13 are commonly associated with CHDs [6, 7]. Though 80% CHDs are sporadic in origin [8], some familial cases of Atrial Septal Defect (ASD), Ventricular Septal Defect (VSD) and Hypoplastic Left Heart Syndrome (HLHS) are recorded but the inheritance patterns are complex [9, 10]. The recurrence risk in off springs of CHD patients varies from 3- 20% depending on the lesion, with slightly higher recurrence in females [11]. Almost one half of the siblings with recurrent lesions in a family have a different lesion suggesting multifactorial etiology and illusive molecular mechanisms [8, 9, 12].

Technological advances now enable study of these developmental defects, thus closing the gap of knowledge between the morphology and genetics [6]. Novel techniques identify regions in the genome on which transcription factors act, driving their target genes and provide new knowledge on CHD development. The T-box transcription factors (*TBX5*) gene is reported to interact with Nk2 Homeobox 5 (*NKX2.5)* and GATA binding protein 4 (*GATA4*) both transcriptional activator of Natriuretic peptide B, which positively regulates the developing heart. The involvement of several well-established cardiac transcription factors that are expressed in cardiogenic plates such as *NKX2.5, GATA4, TBX5*, *TBX20,* Myosin heavy chain 6 *(MYH6),* Actin alpha cardiac muscle (*ACTC1*) and Myocyte enhancer factor 2C (*MEF2C*) have been extensively studied in both human and animal experiments [13-17]. Role of Mutations in transcription factors have been studied for non-syndromic CHDs [18].

Point mutations of cardiac transcription factor genes, single nucleotide polymorphism (SNPs), aneuploidy, and chromosomal copy number variants (CNVs) are directly associated with CHDs. Association of single SNPs seldom lead to complex disease manifestation [19]. There are substantial genetic predispositions to inherited as well as de novo variants with variable effect sizes towards disease risk. Since mutations are rare it requires large numbers to be screened, also same mutations may not be present in all samples. Conventionally a multifactorial inheritance model has been proposed for CHD involving a multitude of susceptibility genes, with low-penetrant common variants or intermediate-penetrant rare variants, superposed on unfavourable environmental factors as causal [15, 20, 21]. Several ethnic or racial differences may also be observed [22]. It is important to investigate genotype–phenotype correlation to provide leads with an opportunity to predict the prognosis.

Limited genetic diagnosis is available for many of the CHDs. Therefore several commercial ventures to sequence genes have been undertaken [23, 24]. These ventures discovered several de novo mutations in the known as well as new genes [6, 12]. Genome wide association studies (GWAS) involve the comparison of genetic variants (known as well as unknown), which can be used to detect genetic risk factors of big and small effect to CHD manifestation [12, 25, 26]. So far more than 500 genes have been estimated with a potential role in the development of CHD [27]. Genetic studies on transcription factors like *GATA4, NKX2.5, TBX1*and *TBX20* have previously shown to identify new mutations in Indian population. [28-33]. Till date limited candidate gene studies have been done in India [34] and only one using exome sequencing [35].

Next generation sequencing (NGS) technologies meet a high standard of evidence and also afford correct predictions in novel datasets. In this study we select association findings from GWAS on CHDs and evaluate in a north Indian cohort. Screening for SNPs and not mutations may reflect better in an association study. Therefore, we chose to assess association of common variants primarily from previous GWAS or meta-analysis studies [26, 36-39] in Caucasians and tested their replicability in our adequately powered study samples.

## Methodology

### Study samples

#### Cases

The study was approved by Institutional Ethics Committee (IEC) at Sri Sathya Sai Sanjeevani Research Foundation (SSSSRF), Palwal, India. Samples were recruited from the Sai Sanjeevani biobank for Congenital Heart of SSSSRF from the period of September 2018 to September 2021. *n* = 306 CHD cases who underwent surgery or cath interventions at Sri Sathya Sai Sanjeevani International Centre for Child Heart Care & Research, Palwal, Haryana were recruited for this study. All methods were performed in accordance with the relevant guidelines and regulations laid down by Indian Council of Medical Research(ICMR). Samples from clinically identified known syndromes or showing distinct extracardiac features were excluded from the study. All the samples selected for the study were non-syndromic based on medical examination and the clinical phenotypes were segregated into cyanotic and acyanotic types. *n* = 108 cyanotic and *n* = 198 acyanotic CHD cases were included. Categorization of cases into 10 subphenotypes (ASD; VSD; TOF: Tetralogy of Fallot; VSD + PS:VSD + Pulmonary Stenosis; DCRV: Double Chamber Right Ventricle; TGA: Transposition of the Great Arteries; SV: Single Ventricle; AVSD: Atrioventricular Septal Defect; TAPVC: both Total and Partial Anomalous Pulmonary Venous Connection; Miscellaneous cases) was accomplished based on intervention procedure, ECHO findings and patient history. Sample distributions are given in Supplementary Table 1.

#### Controls groups

Age matched control group of north India origin was an ideal control for this study but was unavailable and is a limitation of the study. Therefore, we compared it to adequately numbered adults of north Indian origin available in public database [40] (http://asia.ensembl.org/Homo_sapiens/Variation) and published literature [41, 42]. The three groups described below were combined to get a substantial number of controls [43].

Control group1: *n* = 48 adults of north Indian origin unaffected for CHD based on clinical evaluation as well as an ECHO for confirmation were included in the study. Control group2: Since the inhouse control group was small, *n* = 489 open source 1000 Genomes South Asian data was also used as part of reference controls. Control group3: Comparable genotypes for 8 common markers from north India controls in a published study on Celiac Disease [41, 42] was available for *n* = 1170 adults.

#### Marker selection

Assuming large hypothesis-free studies would reflect true associations with disease, most of the GWAS associations were tested in this study. In the absence of contemporary Indian data, associations from Caucasians who are closer to north Indians in ancestry [44], were tested for their contribution. Top 50 markers associated in GWAS catalogue for CHD phenotypes or through maternal influence were shortlisted but only 35 could be accommodated in the assay pool. SNPs having *p* > 10^–6^ and a Minor Allele Frequency(MAF) > 0.001 were selected for inclusion. Priority was given to polymorphic SNPs having a functional implication on the gene product. The study samples had 80% power to detect associations for the SNPs having MAF > 0.1 and an Odds Ratio > 1.5.

#### Experimental method

Samples were isolated by the conventional phenol–chloroform phase separation method. The coded samples were blinded and genotyped through a commercial facility (Genes2Me: https://www.genes2me.com) using Agena MassARRAY technology, which is based on matrix-assisted laser desorption/ionization—time of flight (MALDI-TOF) mass spectrometry. Genetic polymorphisms are distinguished by analysis of their individual mass, excluding the need for fluorescence or labelling. Control samples, duplicates, negatives and positives were used for quality control. Out of 35 SNPs, two failed in assay design and ten SNPs (rs6763159; rs11894932; rs365990; rs17189763; rs2010963; rs350916; rs436582; rs4366490; rs8061121; rs870142) gave ambiguous reads on QC and were removed from the analysis. Only 23 markers and samples which had > 80% genotype calls were retained in the study. A chi-square test was used for association measures and Fisher’s exact test was used, if the expected number was less than five. Statistical tools like SPSS version 21.0 and free online tools [45] (https://vassarstats.net/) were used for analysis. Power was calculated using Quanto [46] (http://biostats.usc.edu/Quanto.html).

## Results

A total of *n* = 23 SNPs in Hardy Weinberg Equilibrium (stringent cut off: *p* > 0.001) were included in the analysis (Supplementary Table 2). Since two SNPs rs2046060 and rs12165908 were monomorphic hence they could not be utilised further for association study. No significant allelic association was seen among cyanotic and acyanotic cases groups. These were still analysed separately vs all controls.

Markers with allelic and genotypic associations are tabulated and presented in Table 1 and 2 for acyanotic, cyanotic and combined categories.

**Table 1:** Allelic association for all analysed SNPs

		SNP details				All Controls Vs Acynanotic	All Controls Vs Cynotic	All Controls Vs Combined cases
Mapped gene	SNP	A1	A2	F_A	F_U	ChiSq; p	ChiSq; p	ChiSq; p
*ENSA*	rs12045807	C	T	0.10	0.06	0.81;0.37	0.35;0.55	0.13;0.72
*CRELD1*	rs73118372	C	T	0.01	0	**10.89;0.001**	**5.71;0.02**	**15.7; < 0.0001**
*SYNPR-AS1, SYNPR*	rs1975649	T	C	0.35	0.46	0.58;0.45	0.13;0.72	0.65;0.42
INTERGENIC	rs185531658	C	T	0.005	0	0.02*	1*	0.04*
*ELN*	rs2071307	A	G	0.25	0.24	1.4;0.24	0.75;0.39	0.19;0.66
*CHD7*	rs3763592	T	C	0.05	0.10	0.11;0.74	0.49;0.48	0.03;0.86
LINC02676	rs2388896	A	G	0.28	0.29	1.27;0.26	2.37;0.12	3.14;0.08
*NRP1*	rs2228638	T	C	0.09	0.07	0.09;0.76	1.22;0.27	0.77;0.38
*UCP2*	rs659366	T	C	0.39	0.44	**5.74;0.02**	0.01;0.92	3.57;0.06
*ATXN2-AS, BRAP*	rs11065987	G	A	0.05	0.02	1.18;0.27	0.02;0.89	0.77;0.38
*SH2B3, PTPN11*	rs11066320	A	G	0.05	0.02	0.38;0.54	0.44;0.51	0.66;0.42
INTERGENIC	rs1497062	A	T	0.33	0.34	0.17;0.68	1.23;0.27	0.09;0.76
*MYH6*	rs28711516	T	C	0.04	0.01	**4.65;0.03**	**9.22;0.002**	**11.18;0.0008**
*MYH7*	rs735712	A	G	0.05	0.06	**7.29;0.01**	**4.98;0.03**	**10.98;0.0009**
*LINC02252, GJD2*	rs6495706	C	G	0.05	0.06	1.64;0.2	0.38;0.54	1.67;0.2
*PCSK6*	rs3784481	G	A	0.49	0.5	0.07;0.79	0.89;0.35	0.5;0.48
INTERGENIC	rs6499100	C	T	0.446	0.489	0.19;0.66	0.05;0.82	0.22;0.64
*PKD1L2*	rs55788414	T	C	0.058	0.035	1.49;0.22	0.19;0.66	1.32;0.25
*GOSR2*	rs11874	A	G	0.02	0	**4.31;0.04**	0.48;0.49	**3.81;0.05**
*JAG1*	rs35761929	C	G	0.153	0.257	0.19;0.66	0.38;0.54	0;1
*MYH7B*	rs3746446	C	T	0.248	0.128	0.17;0.68	0.01;0.92	0.06;0.81

*A1* Allele 1, *A2* Allele 2, *F_A* Frequency of A1 in cases, *F_U* Frequency of A1 in controls, *ChiSq* Pearson’s correlation, *p* Significance

^*^Fishers test p values (in cell counts less than five), All Significant ChiSquare *p* values in **bold** font

**Table 2:** Genotypic Association for all analysed SNPs

		Control**				Cases**			**All Controls Vs Acyanotic cases**	**All Controls Vs Cyanotic cases**	**All Controls Vs Combined cases**
Mapped gene	**SNP**	**Control group 1**	**Control group 2**	**Control group 3#**	**All Control**	**Acyanotic**	**Cyanotic**	**Total cases**	**ChiSq:p**	**ChiSq:p**	**ChiSq:p**
*ENSA*	rs12045807	0/5/35	4/83/402	19/212/930	23/300/1367	6/31/146	1/17/88	7/48/234	4.01;0.13	0.35;0.84	2.02;0.36
*CRELD1* ^*^	rs73118372	0/0/46	5/53/431	-	5/53/477	0/4/150	0/3/89	0/7/239	-	-	-
*SYNPR-AS1, SYNPR*	rs1975649	3/29/6	49/217/223	126/510/516	178/756/745	12/101/65	11/42/40	23/143/105	**9.41;0.01**	0.16;0.92	5.74;0.06
INTERGENIC ^*^	rs185531658	0/0/45	0/0/489	-	0/0/534	0/3/180	0/0/103	0/3/283	-	-	-
*ELN*	rs2071307	1/15/19	19/186/284	64/436/643	84/637/946	10/74/90	3/36/59	13/110/149	1.62;0.44	0.98;0.61	0.49;0.78
*CHD7* ^*^	rs3763592	0/9/35	1/43/445	-	1/52/480	0/16/157	0/13/90	0/29/247	-	-	-
LINC02676	rs2388896	4/16/21	52/208/229	115/496/528	171/720/778	9/71/75	8/32/51	17/103/126	3.16;0.21	3.09;0.21	3.47;0.18
*NRP1*	rs2228638	0/7/41	10/109/370	15/186/955	25/302/1366	1/34/147	0/17/89	1/51/236	1.08;0.58	-	2.44;0.29
*UCP2*	rs659366	3/26/7	62/239/188	122/518/504	187/783/699	25/90/55	8/56/39	33/146/94	**6.24;0.04**	2.56;0.28	5.46;0.07
*ATXN2-AS, BRAP*	rs11065987	0/2/45	4/51/434	-	4/53/479	1/14/174	1/10/99	2/24/273	1.14;0.57	0.09;0.95	0.82;0.66
*SH2B3, PTPN11*	rs11066320	0/2/45	5/54/430	-	5/56/475	2/16/171	1/9/100	3/25/271	0.63;0.73	0.52;0.77	0.96;0.62
INTERGENIC	rs1497062	4/19/17	59/215/215	-	63/234/232	27/63/76	9/42/49	36/105/125	3.12;0.21	1.22;0.54	1.7;0.43
*MYH6*	rs28711516	0/1/46	3/79/407	-	3/80/453	1/16/174	0/5/105	1/21/279	5.28;0.07	-	**11.77;0.003**
*MYH7*	rs735712	0/5/37	4/97/388	-	4/102/425	2/15/155	0/10/86	2/25/241	**10.44;0.01**	-	**13.03;0.002**
*LINC02252, GJD2*	rs6495706	0/6/41	2/65/422	-	2/71/463	0/20/175	2/9/100	2/29/275	-	5.14;0.08	2.92;0.23
*PCSK6*	rs3784481	11/18/11	127/238/124	230/575/332	368/831/467	42/75/50	25/48/24	67/123/74	1.59;0.45	0.92;0.63	1.59;0.45
INTERGENIC	rs6499100	9/27/10	86/247/156	204/588/350	299/862/516	35/91/54	21/52/33	56/143/87	0.29;0.87	0.43;0.81	0.52;0.77
*PKD1L2*	rs55788414	0/3/40	1/73/415	-	1/76/455	1/18/164	1/12/95	2/30/259	2.94;0.23	2.28; 0.32	3.86;0.15
*GOSR2* ^*^	rs11874	0/0/46	1/38/450	-	1/38/496	0/6/185	0/6/102	0/12/287	-	-	-
*JAG1*	rs35761929	1/16/18	16/110/363	-	17/126/381	5/43/115	1/24/71	6/67/186	0.37;0.83	1.4;0.5	0.76;0.68
*MYH7B*	rs3746446	0/11/32	33/193/263	-	33/204/295	7/71/97	4/46/55	11/117/152	1.3;0.52	1.68;0.43	2.35;0.31

^*^Association could not be calculated because of very low counts

^**^genotype counts of variant homozygous / heterozygous/wildtype homozygous for each category are denoted

^#^only 8 markers data available; All associated ChiSq: *p* values in **bold** font

Allelic Association: rs73118372 on chr. 3, rs28711516 and rs735712 on chr. 14 exhibited association for all three categories (acyanotic, cyanotic and combined categories; Table 1). rs73118372 in *CRELD1*(χ^2^ = 15.7; *p* < 0.0001); rs28711516 in *MYH6*(χ^2^ = 11.18; *p* = 0.00083) and rs735712 in *MYH7* (χ^2^ = 10.98; *p* = 0.0009) showed strong allelic associations. Variant rs11874 in *GOSR2* had nominal association (χ^2^ = 3.81; *p* = 0.051) and rs185531658 an intergenic SNP on Chr 5 [39] showed association on Fisher’s test as allele counts were low (*p* = 0.043) while rs659366 in *UCP2* and rs2388896 intergenic SNP demonstrated a weak trend of association (Table 1).

All associations were mainly driven by similar association in the larger acyanotic group. For the SNP rs659366 the association strengthen (χ^2^ = 5.74; *p* = 0.017) in the acyanotic group. The cyanotic association was established only for rs73118372, rs28711516 and rs735712.

### Genotypic association

Similar trend was observed for genotypic associations as illustrated in Table 2. rs1975649(χ2 = 5.74; *p* = 0.057) of *SYNPR* on Chr 3; rs28711516 (χ^2^ = 11.75; *p* = 0.0028) and rs735712 (χ^2^ = 13.03; *p* = 0.0015) on Chr 14 showed genotypic association. rs659366 demonstrated a trend on association (χ^2^ = 5.46; *p* = 0.065).

### Associations with CHD subtypes

Seven allelic associations with ASD; three with TOF; two with VSD + PS; one each with TGA and AVSD phenotypes were observed. rs735712 of *MYH7* (*p* = 0.0029) showed strongest association with VSD subtype amongst all associations observed. ASD, VSD and TOF categories were adequately numbered for association analysis. All subtypes except ASD, VSD and TOF were combined for analysis and showed association for rs73118372, rs28711516 and rs735712 (Table 3).

**Table 3:** Allelic Association of CHD subtypes for all analysed SNPs

		**ASD**	**VSD**	**TOF**	**VSD + PS**	**DCRV**	**TGA**	**SV**	**AVSD**	**TAPVC**	**MISC**	**ALL EXCEPT ASD, VSD & TOF**
**Mapped gene**	**SNP**	**ChiSq;p**	**ChiSq;p**	**ChiSq;p**	**ChiSq;p**	**ChiSq;p**	**ChiSq;p**	**ChiSq;p**	**ChiSq;p**	**ChiSq;p**	**ChiSq;p**	**ChiSq;p**
*ENSA*	rs12045807	2.27;0.13	0.01;0.92	0.59;0.44	0.36;0.55	0.27;0.60	0.03;0.86	0.28;0.59	2.78;0.09	0.13;0.72	0.58;0.45	0.10;0.75
*CRELD1*	rs73118372	0.005*	0.07*	**6.14;0.01**	3.07;0.08	1*	0.62*	1*	0.86;0.35	0.39*	0.11*	**6.98;0.008**
*SYNPR-AS1, SYNPR*	rs1975649	**6.99;0.01**	0.09;0.76	0.19;0.66	0.01;0.92	0.51;0.47	0.04;0.84	0.03;0.87	0.27;0.60	2.55;0.11	0.26;0.61	0.17;0.67
INTERGENIC	rs185531658	1*	1*	0.03*	1*	1*	1*	1*	1*	1*	0.05*	0.20*
*ELN*	rs2071307	0.19;0.66	0.02;0.88	0.96;0.33	0.95;0.33	0.35*	0.19;0.66	0.25;0.62	**7.22;0.007**	0.11;0.74	0.09;0.76	0.15;0.69
*CHD7*	rs3763592	2.77;0.09	0.42;0.52	0.26;0.61	3.11;0.08	1*	0.46*	1*	1*	0.63*	0.13;0.72	0.05;0.81
LINC02676	rs2388896	0.08;0.77	0.28;0.59	2.88;0.09	1.09;0.29	0.63;0.43	0.76;0.38	0.53;0.47	1.25;0.26	0.21;0.65	0.93;0.34	2.18;0.14
*NRP1*	rs2228638	0.15;0.69	0.03;0.86	1.55;0.21	0.43;0.51	3.37;0.07	0.003;0.95	0.08;0.78	0.39*	0.32;0.57	0.17;0.68	0.19;0.66
*UCP2*	rs659366	**3.85;0.05**	3.17;0.07	1.1;0.29	0.48;0.49	0.005;0.95	0.08;0.78	0.06;0.81	3.45;0.06	2.72;0.09	1.28;0.26	0.83;0.36
*ATXN2-AS, BRAP*	rs11065987	1.54;0.21	2.10;0.15	0.36;0.55	0.23;0.63	1*	0.05;0.82	0.01;0.92	0.67;0.41	0.39*	0.68;0.41	0.08;0.77
*SH2B3, PTPN11*	rs11066320	1.94;0.16	1.18;0.27	0.69;0.41	0.01;0.92	1*	0.097;0.76	0.0002;0.99	0.49;0.48	0.24;0.63	0.008;0.93	0.18;0.66
INTERGENIC	rs1497062	0.02;0.88	0.29;0.59	0.57;0.45	1.4;0.24	0.001;0.97	1.36;0.24	0.09;0.77	1.10;0.29	0.85;0.35	1.02;0.31	3.56;0.06
*MYH6*	rs28711516	**5.09;0.02**	0.4;0.53	**6.48;0.01**	**7.35;0.007**	0.60;0.44	0.03;0.86	0.63*	0.10;0.75	0.16*	0.72;0.39	**9.29;0.002**
*MYH7*	rs735712	0.8;0.37	0.003*	2.96;0.09	**4.34;0.04**	1*	0.79;0.37	0.15;0.69	0.38*	0.04;0.85	0.72;0.39	**6.48;0.011**
*LINC02252, GJD2*	rs6495706	0.02;0.88	1.04;0.30	3.64;0.06	1.21;0.27	1*	0.15;0.71	0.62*	0.12;0.73	2.18;0.14	0.43;0.51	0.75;0.38
*PCSK6*	rs3784481	0.02;0.88	0.51;0.47	0.02;0.88	0.22;0.64	1.26;0.26	2.66;0.10	0.54;0.46	0.06;0.81	0.09;0.76	2.34;0.13	1.08;0.3005
INTERGENIC	rs6499100	0.53;0.46	1.13;0.29	0.1;0.75	0.51;0.48	1.76;0.19	2.15;0.14	0.98;0.32	0.27;0.60	1.71;0.19	0.94;0.33	0.38;0.54
*PKD1L2*	rs55788414	2.52;0.11	2.93;0.09	0.13;0.72	0.73;0.39	1*	**4.51;0.03**	0.61;0.43	0.39*	0.0014;0.97	0.46;0.49	0.30;0.58
*GOSR2*	rs11874	0.04*	0.09;0.76	2.59;0.11	0.69;0.41	1*	0.04;0.84	1*	1*	0.0008;0.98	0.69;0.40	1.72;0.19
*JAG1*	rs35761929	**4.01;0.05**	0.97;0.32	0.04;0.84	0.31;0.58	0.009;0.92	0.15 8*	0.09;0.76	0.001;0.97	0.04;0.83	0.02;0.89	0.04;0.84
*MYH7B*	rs3746446	**6.69;0.009**	0.13;0.72	0.07;0.79	0.14;0.71	1.90;0.17	0.05;0.82	0.0009;0.98	1.12;0.29	0.002;0.96	0.01;0.92	0.06;0.80

^*^Fishers test *p* values (in cell counts less than five), Misc.: Other Miscellaneous types; All associated ChiSq: *p* values in **bold** font

## Discussion

50% of tested SNPs were substantially associated in either allelic, genotypic or sub-phenotypes of north Indian CHD cohort validating their strong correlation with disease manifestation. Burden of CHD is overall heavy in India [2] and is prominent in north India. Several genetic determinants of this complex developmental disorder have been reported based on conventional candidate genes and contemporary GWAS but mostly in Caucasian populations. However it is very poorly investigated in the ethnically distinct Indian population. This study was an attempt to test the association of Caucasian findings prior to performing a hypothesis-free approach in the study cohort. Of the 23 SNPs which were successfully genotyped in the modest sized study cohort, 11 SNPs showing allelic or genotypic or association with the CHD sub-phenotypes (Table 1, 2, 3) in a trans ethnic population was noteworthy and reiterates the functional relevance of the associated genes/pathways in CHD pathogenesis.

Of the seven associations observed, four SNPs namely rs73118372 (missense variant) Chr 3; rs659366 (promoter region) on Chr 11; rs735712 (synonymous variant) and rs28711516 (missense variant) on Chr 14; are of functional relevance. Strongest allelic association was observed for rs73118372, rs28711516 and rs735712.

The variant rs73118372(c.1136 T > C) in Exon 9 of *CRELD1* is associated with Downs syndrome [47]. *CRELD1* is involved in the formation of atrioventricular cushion [48] and disrupts existing exon splicing, thus altering the protein configuration and making it unstable. It was associated for all cyanotic, acyanotic and combined categories, and with ASD and TOF sub-phenotypes. Intronic SNP rs1975649 (*SYNPR*; Intron2) also on Chr3 exhibited strong association both in allelic and genotypic categories and was also associated with ASD. Cardiac myosin is the molecular motor that powers heart contraction, a property essential for heart function. It also plays a pivotal role in muscle regulation, development, and mechanotransduction [49]. The α-MYHC (*MYH6*) is expressed in atrial muscle and the β-MYHC (*MYH7*) in skeletal slow-twitch muscle and have arisen through a tandem gene duplication event [50] on Chr. 14. The duplication event is not evident in the genomes of other vertebrates (e.g. birds, fish, amphibia) [51]. *MYH6, MYH7* and *MYH7B* are associated with R amplitude [52]. Heterozygous pathogenic variants in *MHY7* have been associated with septal defects or Ebstein anomaly [48] and of *MYH6* with HLHS and cardiac conduction [53, 54]. *MYH6* is associated with non-syndromic coarctation of the aorta [38, 50] and also presented in families of Shone complex [54]. Previous reports on *MYH6* rs28711516 (c.166G > A; p.G56R) associations with atrial fibrillation [53] and sporadic dilated cardiomyopathy [55] and a GWAS study from south India [35] warrant further investigation of this gene. *MYH7* mutations have been reported for Indian families [56, 57]. Exon 12 variant rs735712 (c.1062C > T; p.G354G) has previously been reported in dilated cardiomyopathies in Indian population [58]. Previous linkage study using microarray identified rs1055061in HOMEZ, a ubiquitously expressed transcription factor on the same locus, in 83 consanguineous CHD families from India [59]. In present study, synonymous variant rs3746446 in MYH7B was associated only with ASD phenotype. This SNP showed strong association with congenital cardiovascular left-sided lesions [38]along with rs12045807, a SNP not associated in our study.

Allelic and genotypic association for combined as well as the acyanotic group for promoter variant rs659366 in *UCP2*, having a role in reactive oxygen species (ROS) pathway [60, 61] is already been reported to be associated with maternal diabetes in CHD offsprings [62] and with dietary factors in Asian populations [60]. It was also associated with ASD subtype. rs185531658, a SNP with the strongest association in 4034 patients of CHD [39], was nominally associated both with our combined data and in acyanotic group.

*ELN* rs2071307 showed mild association with AVSD and *PKD1L2* rs55788414 with TGA subtype. *GOSR2* rs11874, a promoter SNP, was detected in patients with anomalies of thoracic arteries and veins, and may affect the expression of *GOSR2* [39]. It showed a marginal association in our population for combined and acyanotic categories. Missense variant rs35761929 in *JAG1*, involved in Notch cell signalling was associated with ASD subtype in present study and was previously found in ten exome sequenced families from India [38]. During development, the notch pathway regulates embryonic cells destiny to be part of the heart, liver, eyes, ears, and spinal column. The Jagged-1 protein continues to play a role throughout life in the development of new blood cells. These four markers reported are too small in number to make a conclusive statement on its role based on the present results and warrant replication in larger sample set.

Findings in the study were predominantly from the coding region and a few from the intergenic region. The *CRELD1, MYH6* and *MYH7* interact with each other during development of the myosin filament, an active and essential component of the heart tissue. Only one of the strongly associated common variants per gene was tested in this study. There may be more rare and common variants associated from these genes and additional association studies are essential to estimate polygenic risk score to make a significant genotype- phenotype risk prediction for CHD.

## Conclusion

*This is a first study testing association of Caucasian GWAS SNPs in a north Indian population.* All the SNPs studied have been previously shown to have a strong role in the development of CHD. 11 out of 21 SNPs were associated in the study cohort and highlighted the role of *CRELD1, MYH6 and MYH7* in non-syndromic CHD. Strong association of markers from Chr3 and Chr14, and with ASD, VSD and TOF sub-phenotypes were notable in this study and warrant replication in independent CHD cohorts of north Indian origin. This may help uncover the mechanism of disease manifestation by a complex landscape of events influenced by variations in genetic, environmental and demographic patterns.

## Supplementary Information

**Additional file 1: SupplementaryTable 1. **Case and Control Sample distribution. **Supplementary Table 2**. Details of Variants selected inthe study [63,64,65,66,67,68,69].

## Data Availability

The data used in this study are available from the corresponding author and could be requested by email. The data are not publicly available due them containing information that could compromise research participant privacy. All SNP information are available at the links: https://www.ncbi.nlm.nih.gov/snp/?term=rs12045807, https://www.ebi.ac.uk/gwas/search?query=rs12045807, https://www.ncbi.nlm.nih.gov/snp/?term=rs73118372, https://www.ebi.ac.uk/gwas/search?query=rs73118372, https://www.ncbi.nlm.nih.gov/snp/?term=rs1975649, https://www.ebi.ac.uk/gwas/search?query=rs1975649, https://www.ncbi.nlm.nih.gov/snp/?term=rs185531658, https://www.ebi.ac.uk/gwas/search?query=rs185531658, https://www.ncbi.nlm.nih.gov/snp/?term=rs2071307, https://www.ncbi.nlm.nih.gov/snp/?term=rs3763592, https://www.ncbi.nlm.nih.gov/snp/?term=rs2388896, https://www.ebi.ac.uk/gwas/search?query=rs2388896, https://www.ncbi.nlm.nih.gov/snp/?term=rs2228638, https://www.ebi.ac.uk/gwas/search?query=rs2228638, https://www.ncbi.nlm.nih.gov/snp/?term=rs659366, https://www.ncbi.nlm.nih.gov/snp/?term=rs11065987, https://www.ebi.ac.uk/gwas/search?query=rs11065987, https://www.ncbi.nlm.nih.gov/snp/?term=rs11066320, https://www.ebi.ac.uk/gwas/search?query=rs11066320, https://www.ncbi.nlm.nih.gov/snp/?term=rs1497062, https://www.ebi.ac.uk/gwas/search?query=rs1497062, https://www.ncbi.nlm.nih.gov/snp/?term=rs28711516, https://www.ncbi.nlm.nih.gov/snp/?term=rs735712, https://www.ncbi.nlm.nih.gov/snp/?term=rs6495706, https://www.ebi.ac.uk/gwas/search?query=rs6495706, https://www.ncbi.nlm.nih.gov/snp/?term=rs3784481, https://www.ncbi.nlm.nih.gov/snp/?term=rs6499100, https://www.ebi.ac.uk/gwas/search?query=rs6499100, https://www.ncbi.nlm.nih.gov/snp/?term=rs55788414, https://www.ebi.ac.uk/gwas/search?query=rs55788414, https://www.ncbi.nlm.nih.gov/snp/?term=rs11874, https://www.ebi.ac.uk/gwas/search?query=rs11874, https://www.ncbi.nlm.nih.gov/snp/?term=rs35761929, https://www.ncbi.nlm.nih.gov/snp/?term=rs3746446, https://www.ebi.ac.uk/gwas/search?query=rs3746446

## Abbreviations

*ACTC1*: Actin alpha cardiac muscle
ASD: Atrial septal defect
*ATXN2-AS, BRAP*: Ataxin antisense RNA, *BRCA 1*(Breast cancer gene1) associated protein
AVSD: Atrioventricular septal defect
CHARGE: Coloboma, Heart defects, Atresia choanae, growth Retardation, Genital abnormalities, and Ear abnormalities
CHD: Congenital heart defect
*CHD7*: Chromodomain helicase DNA binding protein 7
Chr.: Chromosome number
CNV: Copy number variation
*CRELD1*: Cysteine rich with EGF like domains 1
DCRV: Double chamber right ventricle
*ELN*: Elastin
*ENSA*: Endosulfine alpha
*GAS1*: Growth arrest-specific 1
*GATA4*: GATA binding protein 4
*GOSR2*: Golgi SNAP receptor complex member 2
GWAS: Genome-wide association studies
HLHS: Hypoplastic left heart syndrome
*ISLET1*: ISL1 transcription factor LIM/homeodomain
*JAG1*: Jagged canonical Notch ligand 1
LINC02252: Long intergenic non-protein coding RNA 2252
*GJD2*: Gap junction protein delta 2
LINC02676: Long intergenic non-protein coding RNA 2676
*MEF2C*: Myocyte enhancer factor 2c
MLC: Myosin light chains
*MYH6*: Myosin heavy chain 6
*MYH7*: Myosin heavy chain 7
*MYH7B*: Myosin heavy chain 7B
*NKX2.5*: Nk2 homeobox 5
*NRP1*: Neuropilin 1
*OSR1*: Odd-skipped-related transcription factor 1
*PCSK6*: Proprotein convertase subtilisin/kexin type 6
*PKD1L2*: Polycystin 1 like 2
PS: Pulmonary stenosis
*PTPN11*: Tyrosine-protein phosphatase non-receptor type 11
*SH2B3*: SH2B (Src homology 2B) adaptor protein 3
SNP: Single nucleotide polymorphism
SV: Single ventricle
*SYNPR-AS1*, SYNPR: Synaptoporin antisense RNA 1
TAPVC: Total anomalous pulmonary venous return
TBX: T-box transcription factors
TGA: Transposition of the great arteries
*UCP2*: Uncoupling protein 1; VSD: Ventricular septal defect
