# Supplementary material for: Case–control association study of congenital heart disease from a tertiary paediatric cardiac centre from North India
Source: BMC Pediatr. 2023 Jun 15;23:290. doi: 10.1186/s12887-023-04095-x (PMC10268439; doi:10.1186/s12887-023-04095-x)
Supplement: Supplementary file 1 — Additional file 1: SupplementaryTable 1. Case and Control Sample distribution. Supplementary Table 2. Details of Variants selected inthe study [63,64,65,66,67,68,69]. [file 12887_2023_4095_MOESM1_ESM.doc]

Supplementary Table 1: Case and Control Sample distribution

| **Samples** | **Gender** | |  |
| --- | --- | --- | --- |
|  | **males** | **females** | **total** |
| **Cases** | | | |
| ASD | 18 | 16 | 34 |
| VSD | 78 | 44 | 122 |
| TOF | 54 | 13 | 67 |
| VSD+PS | 5 | 0 | 5 |
| TGA | 7 | 4 | 11 |
| SV | 3 | 5 | 8 |
| AVSD | 5 | 5 | 10 |
| TAPVC | 11 | 3 | 14 |
| All OTHERS | 18 | 17 | 35 |
| **TOTAL** | **199** | **107** | **306** |
| **Controls** | | | |
| Lab control | 34 | 14 | 48 |
| BKT controls (personal communication) | 699 | 471 | 1170 |
| SAS controls (public domain) | NA | NA | 489 |

Supplementary Table 2: Details of Variants selected in the study

| **Chr: Location** | **Va0riant and risk allele** | **Type** | **P-Value** | **Mapped Gene** | **Reported trait** | **RAF** | **OR** | **CI [95%]** | **Other Phenotype** | **[Ref]Title** |
| --- | --- | --- | --- | --- | --- | --- | --- | --- | --- | --- |
| 1:150612358 | rs12045807-C | Intron variant | 9 x 10^-7^ | *ENSA* | Congenital left-sided heart lesions | 0.089 | 1.61 | - |  | [38](A genome-wide association study of congenital cardiovascular left-sided lesions shows association with a locus on chromosome 20) |
| 3:9943989 | rs73118372 | Missense variant |  | *CRELD1* | AV canal defect, TOF |  |  |  |  | [47](Polymorphic haplotypes of CRELD1 differentially predispose Down syndrome and euploids individuals to atrioventricular septal defect) |
| 3:63453210 | rs1975649-? | Intron variant | 3 x 10^-7^ | *SYNPR-AS1, SYNPR* | Congenital left-sided heart lesions | 0.33 | 1.65 | [1.36-2.01] | LncRNA | [37](Genome-wide association study of maternal and inherited effects on left-sided cardiac malformations) |
| 5:113800824 | rs185531658-? | Intergenic variant | 5 x 10^-9^ | *-* | Congenital heart disease | - | 2.16 | [1.67-2.80] |  | [39](Congenital heart disease risk loci identified by genome-wide association study in European patients) |
| 7:74056384 | rs2071307 | Missense variant |  | *ELN* | Supravalvar aortic stenosis |  |  |  | Cutis laxa | [48](Update in Biomolecular and Genetic Bases of Bicuspid Aortopathy) |
| 8:60852714 | rs3763592 | Splice region variant |  | *CHD7* | CHARGE syndrome |  |  |  | Hypogonadotropic Hypogonadism 5 with or without anosmia | [49](Complete screening of 50 patients with CHARGE syndrome for anomalies in the CHD7 gene using a denaturing high-performance liquid chromatography-based protocol: new guidelines and a proposal for routine diagnosis) |
| 10:8912261 | rs2388896-G | Intron variant | 9 x 10^-8^ | *LINC02676* | Tetralogy of Fallot | 0.4** | 1.28 | [1.15-1.43] | lncRNA | [36](Genome-wide association study identifies loci on 12q24 and 13q32 associated with tetralogy of Fallot) |
| 10:33186354 | rs2228638-A | Missense variant | 2 x 10^-7^ | *NRP1* | Tetralogy of Fallot | 0.096 | 1.45 | [1.239-1.69] |  | [36] (Genome-wide association study identifies loci on 12q24 and 13q32 associated with tetralogy of Fallot) |
| 11:73983709 | rs659366 | Non-coding transcript exon variant |  | *UCP2* |  |  |  |  | BMI, Type 2 diabetes | [50](Association and interaction effect of UCP2 gene polymorphisms and dietary factors with congenital heart diseases in Chinese Han population) |
| 12:111634620 | rs11065987-G | Intergenic variant | 8 x 10^-11^ | *ATXN2-AS, BRAP* | Tetralogy of Fallot | 0.418 | 1.34 | [1.208-1.496] | cholestrol, BMI, HB | [36](Genome-wide association study identifies loci on 12q24 and 13q32 associated with tetralogy of Fallot) |
| 12:112468611 | rs11066320 | Intron variant |  | *SH2B3, PTPN11* | Cardiometabolic disease |  |  |  | cholesterol, systolic BP, rheumatoid arthritis | [51](The genetic architecture of plasma kynurenine includes cardiometabolic disease mechanisms associated with the SH2B3 gene) |
| 13:82007307 | rs1497062-? | Intergenic variant | 5 x 10^-6^ | *-* | Atrioventricular septal defects in Down syndrome | NR | 1.95 | [1.46-2.59] | Down syndrome | [52](Genome-Wide Association Study of Down Syndrome-Associated Atrioventricular Septal Defects) |
| 14:23407058 | rs28711516 | Missense variant |  | *MYH6* | Abnormality of cardiovascular system, familial hypertrophic 14cardiomyopathy |  |  |  |  | [53]( Multi-ancestry GWAS of the electrocardiographic PR interval identifies 202 loci underlying cardiac conduction) |
| 14:23429851 | rs735712 | Synonymous variant |  | *MYH7* | Cardiomyopathy |  |  |  |  | [54](Novel Mutations in Î²-MYH7 Gene in Indian Patients With Dilated Cardiomyopathy) |
| 15:34684669 | rs6495706-? | Intergenic variant | 9 x 10^-6^ | *LINC02252, GJD2* | Congenital left-sided heart lesions | 0.06** | 2.13 | [1.52-3.03] |  | [37](Genome-wide association study of maternal and inherited effects on left-sided cardiac malformations) |
| 15:101391959 | rs3784481 | Intron variant |  | *PCSK6* |  |  |  |  |  | [55](Genome-wide linkage analysis of congenital heart defects using MOD score analysis identifies two novel loci) |
| 16:52797550 | rs6499100-A | Intergenic variant | 1 x 10^-6^ | *-* | Tetralogy of Fallot | 0.519 | 1.26 | [1.14-1.4] | lncRNA | [36](Genome-wide association study identifies loci on 12q24 and 13q32 associated with tetralogy of Fallot) |
| 16:81151334 | rs55788414-? | Intron variant | 6 x 10^-8^ | *PKD1L2* | Left ventricular obstructive tract defect (maternal effect) | NR | 5.81 | [3.082993-10.969667] |  | [26](Genome-Wide Association Studies and Meta-Analyses for Congenital Heart Defects) |
| 17:46939827 | rs11874 | 3 prime utr variant |  | *GOSR2* | Congenital heart disease anomalies of thoracic arteries and veins |  |  |  | Progressive myoclonic epilepsy | [39](Congenital heart disease risk loci identified by genome-wide association study in European patients) |
| 20:10641853 | rs35761929 | Missense variant |  | *JAG1* | Alagille syndrome 1, Abnormality of cardiovascular system, Isolated non-syndromic CHD |  |  |  |  | [56](JAG1 Mutation Spectrum and Origin in Chinese Children with Clinical Features of Alagille Syndrome) |
| 20:34986962 | rs3746446-G | Synonymous variant | 1 x 10^-8^ | *MYH7B* | Congenital left-sided heart lesions | 0.18 | 1.23 | - |  | [38](A genome-wide association study of congenital cardiovascular left-sided lesions shows association with a locus on chromosome 20) |

** Opposite allele
